# Supplementary material for: A high-resolution mRNA expression time course of embryonic development in zebrafish
Source: eLife. 2017 Nov 16;6:e30860. doi: 10.7554/eLife.30860 (PMC5690287; doi:10.7554/eLife.30860)
Supplement: Supplementary file 6. [file elife-30860-supp6.zip › biolayout-clusters-files/Cluster082-genes.html]

Cluster082


# Cluster082: Genes

| Ensembl ID | Gene Name | Chr | Start | End | Biotype |
| --- | --- | --- | --- | --- | --- |
| ENSDARG00000102332 | ENSDARG00000102332 | KN149966.1 | 102744 | 131059 | protein\_coding |
| ENSDARG00000103962 | add1 | 21 | 1964471 | 2042174 | protein\_coding |
| ENSDARG00000056803 | ap1s1 | 7 | 25973869 | 25992013 | protein\_coding |
| ENSDARG00000095086 | cfdp1 | 18 | 22426985 | 22497701 | protein\_coding |
| ENSDARG00000056141 | coq10b | 9 | 32367628 | 32377699 | protein\_coding |
| ENSDARG00000078233 | ctnnd1 | 1 | 43471897 | 43527762 | protein\_coding |
| ENSDARG00000002128 | cwf19l1 | 4 | 17653755 | 17664590 | protein\_coding |
| ENSDARG00000037846 | hm13 | 23 | 467630 | 480591 | protein\_coding |
| ENSDARG00000006408 | hmgb3b | 21 | 34085269 | 34095286 | protein\_coding |
| ENSDARG00000059853 | manbal | 6 | 55203116 | 55209049 | protein\_coding |
| ENSDARG00000104130 | rbm26 | 6 | 4211040 | 4241150 | protein\_coding |
| ENSDARG00000104114 | setd8b | 5 | 69013438 | 69021245 | protein\_coding |
| ENSDARG00000015946 | slc44a1a | 1 | 51998629 | 52017623 | protein\_coding |
| ENSDARG00000007405 | strap | 4 | 16887855 | 16894075 | protein\_coding |
| ENSDARG00000030981 | tmem127 | 8 | 4747639 | 4757402 | protein\_coding |
